# Design, Fabrication, Testing and Simulation of a Rotary Double Comb Drives Actuated Microgripper

**DOI:** 10.3390/mi12101263

**Published:** 2021-10-17

**Authors:** Nicola Pio Belfiore, Alvise Bagolini, Andrea Rossi, Gabriele Bocchetta, Federica Vurchio, Rocco Crescenzi, Andrea Scorza, Pierluigi Bellutti, Salvatore Andrea Sciuto

**Affiliations:** 1 Department of Industrial, Electronic and Mechanical Engineering, Roma Tre University, 00146 Rome, Italy; andrea.rossi@uniroma3.it (A.R.); gab.bocchetta@stud.uniroma3.it (G.B.); federica.vurchio@uniroma3.it (F.V.); andrea.scorza@uniroma3.it (A.S.); salvatore.sciuto@uniroma3.it (S.A.S.); 2 Micro Nano Facility, Fondazione Bruno Kessler, 38123 Trento, Italy; bagolini@fbk.eu (A.B.); bellutti@fbk.eu (P.B.); 3 Department of Information Engineering, Electronics and Telecommunications Sapienza University of Rome, 00184 Rome, Italy; rocco.crescenzi@uniroma1.it

**Keywords:** microgripper, comb drives, DRIE, MEMS, compliant mechanisms

## Abstract

This paper presents the development of a new microgripper actuated by means of rotary-comb drives equipped with two cooperating fingers arrays. The microsystem presents eight CSFH flexures (Conjugate Surface Flexure Hinge) that allow the designer to assign a prescribed motion to the gripping tips. In fact, the adoption of multiple CSFHs gives rise to the possibility of embedding quite a complex mechanical structure and, therefore, increasing the number of design parameters. For the case under study, a double four-bar linkage in a mirroring configuration was adopted. The presented microgripper has been fabricated by using a hard metal mask on a Silicon-on-Insulator (SOI) wafer, subject to DRIE (Deep Reactive Ion Etching) process, with a vapor releasing final stage. Some prototypes have been obtained and then tested in a lab. Finally, the experimental results have been used in order to assess simulation tools that can be used to minimize the amount of expensive equipment in operational environments.

## 1. Introduction

Microgrippers have been extensively studied for the last two decades as promising tools for a large variety of applications [1,2,3]. A recent paper reviewed about a hundred different microsystems from structural [4] and operational [4] viewpoints.

Some design methods make use of topology optimization [5] or perturbation-based configuration [6], while a more common approach consists of the adoption of a lumped compliance structure obtained by the rigid body replacement method [7]. This approach allows designers to use the pseudo rigid body equivalent mechanism (PRBM) [8,9], which gives rise to the opportunity to use topology [10], planarity [11], and kinematic synthesis [12] to optimize the desired layout of the structure.

After the design, many different methods can be successfully used to fabricate microgrippers [13,14,15,16]. Furthermore, new materials with high biocompatibility have also been investigated [17]. In the present investigation, an approach based on MEMS Technology has been used and described. Then, testing, as the necessary natural stage of validation [18,19], has been carried out on the new microsystems, and finally, a simulation has been built, as a means to optimize the experimental campaigns and to decrease the number of experiments [20,21,22].

The capability of microgrippers has been recently proved in operations of versatile grasping [23] and autonomous pick-and-place [24]. In recent years, several studies investigated the dynamic response of cells subjected to mechanical vibrations. Some of them [25,26,27,28,29,30] demonstrated the effectiveness of using mechanical vibration in cancer research and regenerative medicine. For instance, pure mechanical vibration, without the addition of magnetic particles or exposure to a magnetic field, can induce apoptosis of cancer cells and also enhance the cell absorption of increasing nutrient amounts (such as glucose). Their depletion can eventually lead to programmed cell death. Another application concerns the regenerative medicine, where a detailed evaluation of cell cultures by image analysis revealed that vibration enhances cell growth and differentiation. Mesenchymal stem cells (MSCs) are capable of differentiating into any type of mesenchymal tissue, including bone, cartilage, muscle, and fat. Other papers [27,28,29] investigate the effect of vibration on MSCs as a function of frequency, acceleration, and duration of stimulation. Therefore, the evaluation of the dynamic behavior of a microgripper could be appealing to determine whether it can be exploited to stimulate biological tissues or cells through mechanical vibrations.

Recently, microgrippers equipped with Conjugate Surface Flexure Hinges (CSFH) [31] successfully attempted some operations of grasping and releasing Agarose beans in water drops [32]. More experimental work and simulations have recently shown that the total number of flexures is related to the energy required to deform the structure from neutral to working configurations. Therefore, microgrippers with only one CSFH pair, operated by one pair of rotary-comb drives [33], presented, at the same applied voltage, a range of motion wider than those embedding several CSFHs, as, for example, planar 3-DoF microstages [34] or four-bar linkage microgrippers with bidirectional comb drives [35]. In the latter microsystems, comb drives are arranged in such a way that it is possible to induce either clockwise or counterclockwise jaw rotations. Contrarily, the present paper investigates the possibility of increasing the driving torque applied to the moving links, up to twice as much, by arranging the rotary-comb drives configuration in such a way that they cooperatively work together. In other words, the idea consists of changing the finger arrays configuration from the bidirectional [35] to the mono-directional arrangement with two cooperating drives.

This paper presents the full development of this device from the stages of design to fabrication, dynamic testing, and simulation.

## 2. Design

**Figure 1:**
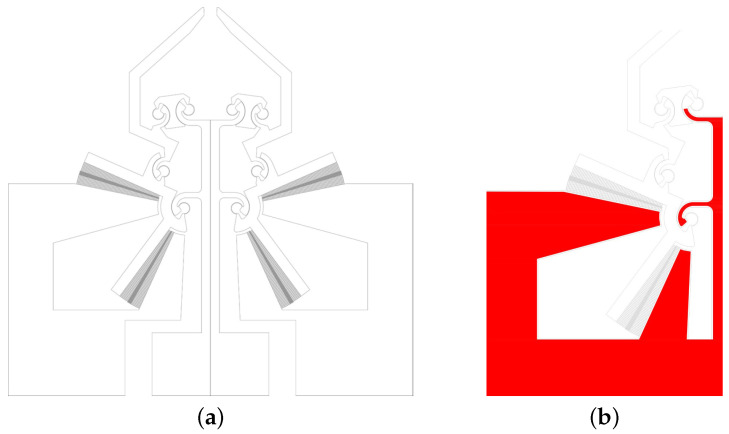
The geometry of the device, including the comb drives, the anchored parts, the compliant linkages, and the jaws (**a**); detailed view of the left-hand side anchored layer (**b**).

The design of a MEMS device follows a symbiotic development between the device concept and the technological processes that will be used for its realization. A deviced based on MEMS Technology is characterized by maximum dimensions spanning from a few millimeters down to a few micrometers for some of its parts. Therefore, the miniaturization involves different behaviors for the material properties used and consequently also in the electro-mechanical behavior of the parts. On the other hand, it is well known that the smaller the size, the more critical technological issues arise. The object of this work has been designed to be a monolithic microgripper capable of obtaining considerable roto-translational displacements of some of its parts through electro-capacitive actuation (comb-drive). Figure 1a shows the basic module used for the design of the photolithographic mask in order to obtain the device. This module includes all parts of the microgripper: tips, CSFHs, comb-drives, and pads.

The grasping task is characterized by the type of motion of the the jaws while they are approaching the target object. The most simple layout consists of a pair of symmetric rotating jaws. However, this configuration is rather constraining because the approaching motion to the target is predetermined, namely a simple rotation. Considering the realm of plane mechanisms, the moving links that are adjacent to the frame link present a rotation about a fixed axis. This can be a very limiting constraint to the functional design. For this reason, many grippers have been designed as a compliant mechanism whose pseudo-rigid body equivalent mechanism (PRBM) [8] is a 4-bar linkage. This solution has been the preferred choice for the present investigation because by using 4-bar linkage type PRBM, the coupler-link, which the jaw is attached to, does not have a fixed rotation axis, whereas this instantaneous rotation axis can be positioned by the designer.

The design requirements suggested the use of monocrystalline silicon as a structural material since it offers mechanical and electrical properties with very good performance. Moreover, monocrystalline silicon shows, at the millimeter scale, a higher elasticity and a lower fragility than that which it presents at the macroscopic scale. The choice of monocrystalline silicon also identifies the type of substrate that will be used: silicon wafer. Furthermore, the need of a material with low electrical resistance and, at the same time, a high mechanical stiffness has oriented the choice towards the SOI (Silicon-On-Insulator) wafer. An SOI wafer with the following characteristics has been the preferred choice to comply with the design requirements:

- 40 μm thick device layer doped to obtain low electrical resistance;
- 3–4 μm silicon oxide layer (BOX);
- handle layer with a thickness of 400 μm to give a high robustness to the device, which is necessary both for its implementation and its manipulation by the operators.

Figure 1b shows the geometry (left side) provided to the handle layer for the strengthening of the device (red areas) and represents the basic module for the photoligraphic mask used to obtain the desired shape for the handle layer by the etching process. It should be noted that the strengthening of the structure is foreseen in the parts that do not have any motion during the actuation, such as the pads for the electrical contacts and the central support structure for the CSFH.

The ideal process for the “bulk micromachining” necessary to obtain this geometric shape is Deep Reactive Ion Etching (DRIE) because it uses an etching that gives rise to a high ratio of the etching gap to the possible depth. In fact, this application needs at least a 40 μm etching for the device layer and a 400 μm etching for the handle layer.

**Figure 2:**
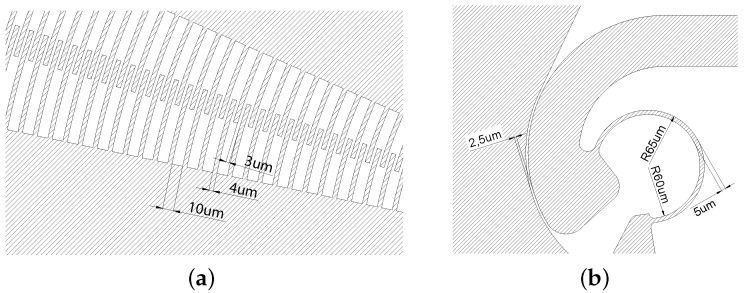
A detailed view of the interdigitated area of the comb drives (**a**) and of the CSFH (**b**).

The device structure is characterized by elements with very different dimensions, and, among these, some elements represent a constraint to functionality. In fact, from the point of view of capacitive electrostatic actuation, it is of fundamental importance that the comb-drive fingers and gaps between the stator and rotor finger arrays have dimensions of a few micrometers (see Figure 2a).

From the kinematic point of view, it is necessary that the curved beams and the gap in the CSFH have also dimensions of a few micrometers, as depicted in Figure 2b.

As the selected technological process (DRIE) is expected to yield a device with both moving and fixed parts, it is of primary importance to manage the silicon etching in order to reach the silicon oxide layer (etching stop layer) in a uniform manner. In fact, the presence of etching areas of different sizes causes the loading effect for the DRIE: with the same process time, the etching is deeper in the large etching opening windows than in the narrow ones.

**Figure 3:**
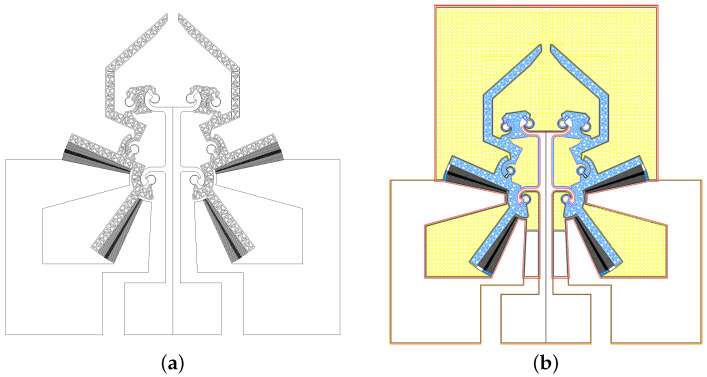
The modified reticular geometry of the suspended parts of the compliant structure (**a**) and the mask adopted for the technological process (**b**).

Since the moving parts must be released from the other layers, it is necessary to remove the silicon oxide. However, the underetching, which has limitations due to the micrometric dimensions of the gap, gives rise to the condensation of the etching gas and therefore the collapse of the upper surface on the lower one (stitching effect), stopping the etching process. In order to avoid both of the above-mentioned problems, all the suspended surfaces were modified into reticulated frames, as shown in Figure 3a. This solution has the advantage of lightening the structure, leaving stiffness almost unchanged, and permits the etchants to reach all the areas that need the underetching. Furthermore, the internal grid has been made in such a way that the thickness of the beams is comparable with the smaller elements’ dimensions in the device.

Figure 3b shows the base module realized for the etching of the substrate layer device. Referring to the figure, the yellow areas are sacrificial platforms whose insertion is necessary to avoid the loading effect. Furthermore, for these platforms, the criterion used for the truss frame is the one followed for the moving parts (blue color).

Quantitative information concerning the adopted comb-drives and CSFH flexures are provided in Table 1. The influence of such quantities on the operational capabilities of this kind of microgripper has been extensively studied in 2018 [36].

## 3. Fabrication

The microgripper was fabricated on 6” SOI wafers by silicon deep reactive ion etching with aluminum masking. The fabrication process was performed using a previously reported sequence [37], but the final release was changed to enable a better performance using HF vapor etching.

The fabrication steps can be summarized as follows.

The masking layers were initially deposited on both the front and back side in the following sequence: TEOS Silicon Oxide (200 nm thick), magnetron-sputtered Aluminum (150 nm thick), and PECVD Silicon Oxide (300 nm thick). The need for a PECVD layer on top of the aluminum layer was previously described in detail in 2019 [38].

The layers were then patterned with a stepper photolithography process and etched in plasma etching to expose the underlying silicon.

The exposed silicon was then etched starting from the front side with deep reactive ion etching (DRIE). Both front and back etchings proceeded down to the buried silicon oxide that is embedded in the SOI wafers and acts as an etch stop.

Last, the devices were released by removing the buried silicon oxide with an HF vapor etching tool (SPTS Primaxx^®^ uEtch, 2021 SPTS Technologies Ltd., Ringland Way, Newport, NP18 2TA, UK).

**Figure 4:**
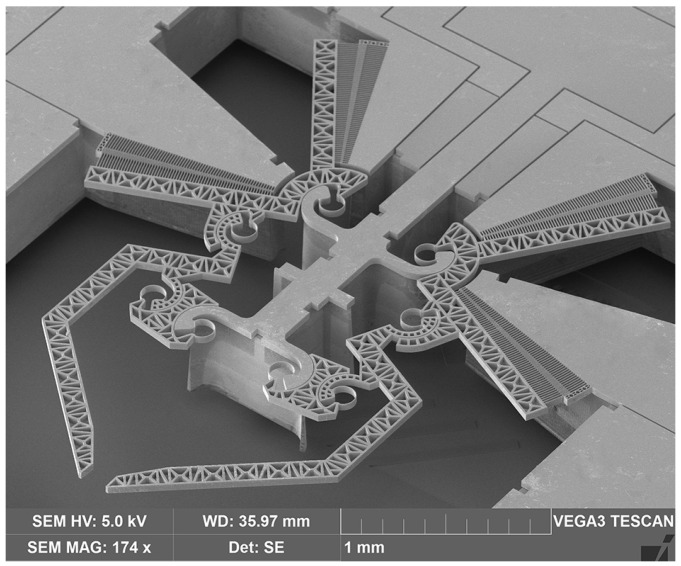
A SEM view of a prototype of the newly developed microgripper with double actuation.

**Figure 5:**
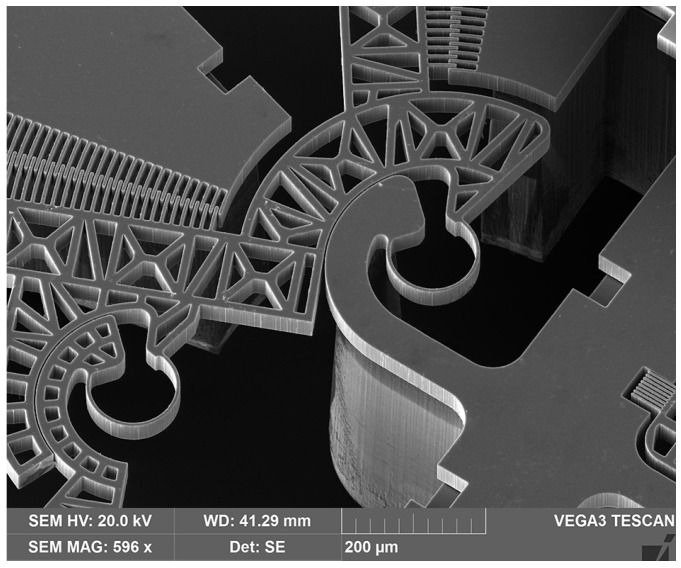
A detailed SEM view of a pair of cooperating combs rotating around the same CSFH: the first member of the CSFH flexure hinge is anchored, while the other one is driven by the combs pair.

After all the steps are completed, the prototype is obtained, as shown in Figure 4. One of the main characteristics of the device consists of the two cooperating array arrangements in the comb drives, with a pair of arrays per side. Considering the left-hand side, Figure 5 shows a detailed view of one pair of arrays, rotating around the same CSFH.

## 4. Testing

Previous studies that concerned the functional characterization of microgrippers showed that these devices exhibit a non-linear behavior since the angular displacement of this kind of capacitive electrostatic actuators is a quadratic function of the supply voltage [32,39,40,41,42,43]. The approach used in the previous studies focused on the static characterization of the microgrippers, where the Device Under Test (DUT) was powered with a direct voltage from 0 V to about 24 V and observed by means of a trinocular optical microscope equipped with a digital camera for image acquisition. In particular, the acquired images have been processed through an automatic software developed by the authors in a MATLAB environment for the measurement of the angular displacement of the comb-drives. More recently, in [44], a novel measurement method based on a marker tracking algorithm has been proposed for the measurement of the angular displacements of the comb-drive, together with the corresponding displacements of the grippers: this approach was found suitable for the evaluation of the dynamic behavior of the DUT powered with 20 V${}_{p-p}$ sinusoidal voltages at different frequencies.

**Figure 6:**
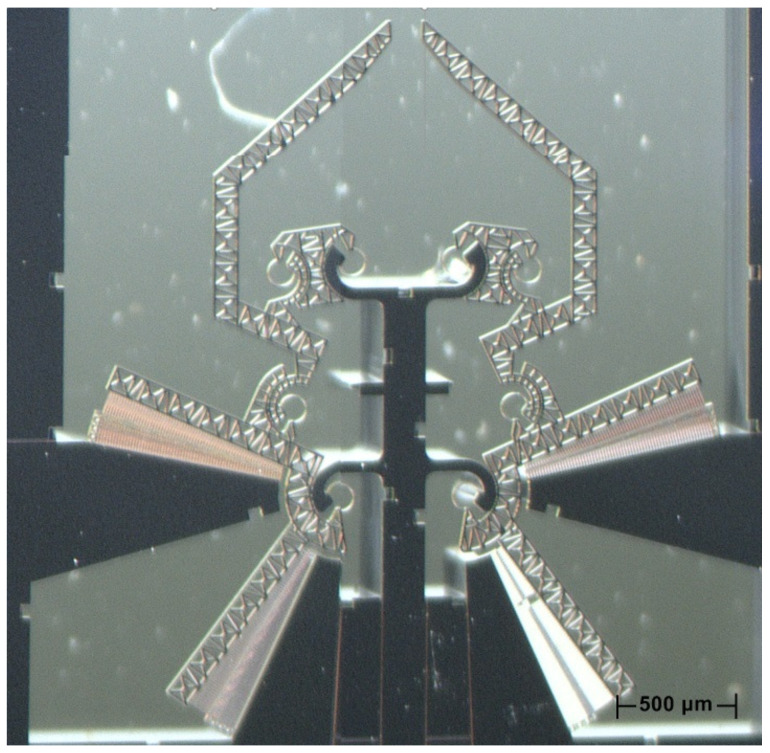
An optical view of a microgripper prototype (DUT).

The above-mentioned experimental approach has been applied in this study for monitoring the dynamic behavior of the comb-drives in the novel prototype (DUT depicted in Figure 6) that has been actuated by providing a sinusoidal voltage signal at different frequencies. Measurements of the comb-drive displacements, velocities, and accelerations have been carried out by means of video acquisition, processing, and analysis procedure implemented in a MATLAB©environment. The following sections will illustrate the experimental setup, the developed video processing software, the uncertainty analysis (to estimate the quality of the measurement results), and, finally, the comparison between the experimental outcomes and numerical results from the simulations.

### 4.1. Experimental Setup

**Figure 7:**
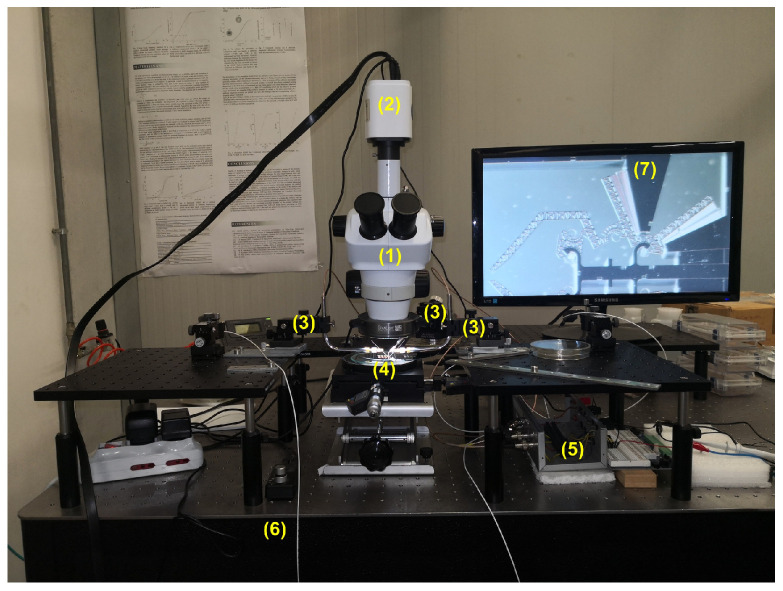
Experimental setup. Optical microscope (1), embedded camera for video acquisition (2), three micropositioners with two embedded probe arms and tungsten needles (3), DUT microgripper prototype (4), protection circuit (5), pneumatic suspension table (6), and display (7).

**Figure 8:**
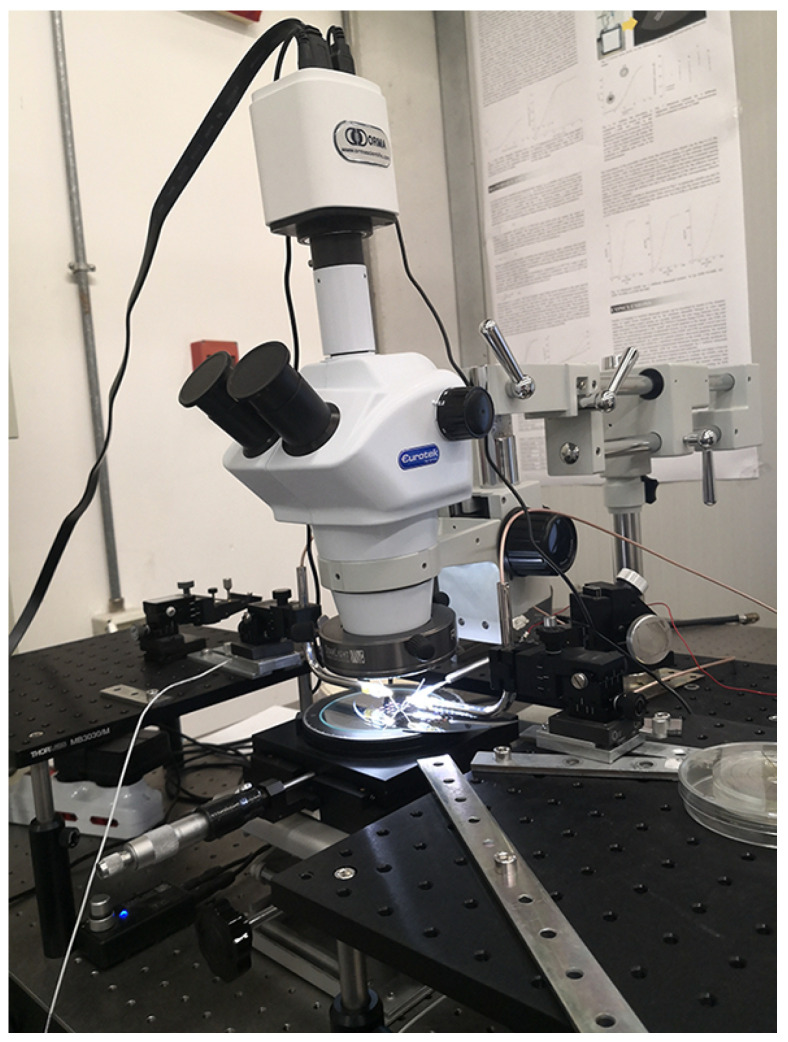
Trinocular optical microscope embedded with a 6 MP digital camera for video acquisition.

**Figure 9:**
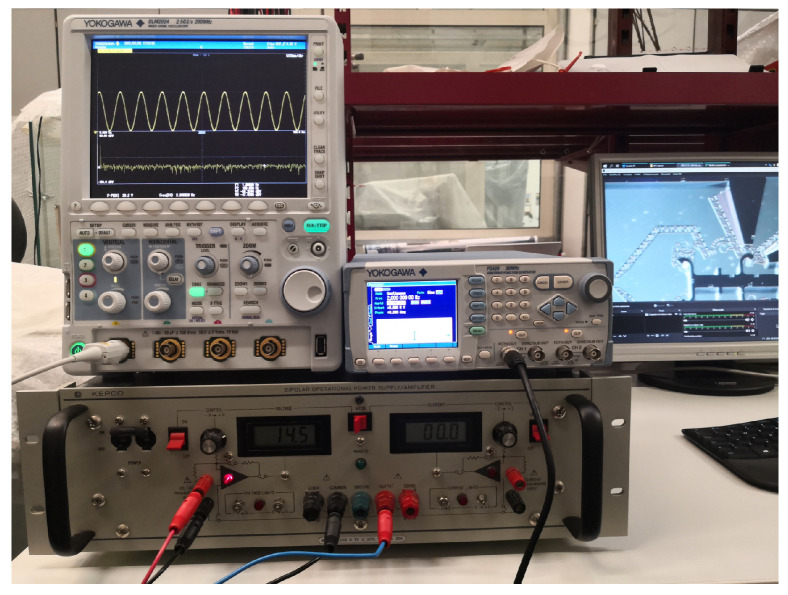
YOKOGAWA FG420 function generator and KEPCO BOP 20-20D power amplifier.

**Figure 10:**
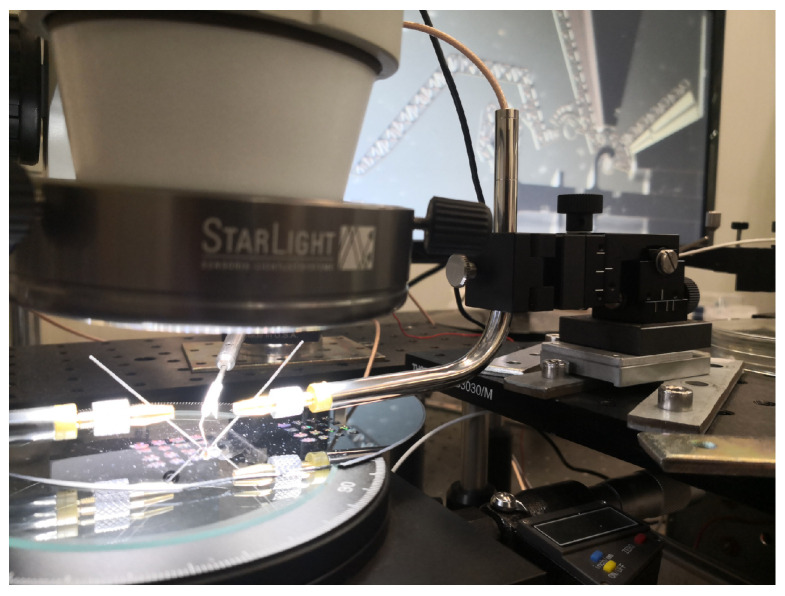
Three tungsten needles connected to the micro-positioners in order to power the device.

The developed experimental setup supplies power to the DUT, captures videos, and processes the acquired data, thanks to an integrated in-house software (Figure 7). The selected microgripper has been positioned under a trinocular optical microscope NB50TS (Figure 8) equipped with a MD6iS digital camera, then the DUT has been powered with a sinusoidal voltage from a YOKOGAWA FG420 function generator and amplified by a KEPCO BOP 20-20D power amplifier (Figure 9); the electrical connection between the DUT and the voltage source is provided by three tungsten needles fixed to three micro-positioners (Figure 10), which provide motion along three orthogonal directions (x, y, z). A protection circuit has been placed between the DUT and the power supply to limit the current in the prototype under examination. All measurements have been conducted on a pneumatic suspension table to keep the whole experimental setup as stable as possible and to limit the environmental vibrations. Videos have been collected at a 60 fps frame rate for different frequencies of the supply voltage, namely 0.5 Hz, 1.0 Hz, 1.5 Hz, 2.0 Hz, 3.0 Hz, and 4.0 Hz. Furthermore, different acquisition time intervals have been set in order to reduce the computational costs on the video processing phase with negligible information loss: at least 70 s, 40 s, 20 s, 15 s, 10 s, or 5 s have been chosen, respectively, for 0.5 Hz, 1.0 Hz, 1.5 Hz, 2.0 Hz, 3.0 Hz, or 4.0 Hz to acquire at least 30 periods of the measured output signal.

The main components of the experimental setup are shown in Figure 7 and reported in Table 2.

### 4.2. Video Processing

The processing and analysis of the collected videos have been carried out by an automatic software implemented in a MATLAB${}^{©}$ environment [44]. The displacement of the comb-drive has been measured by means of a marker-tracking-based algorithm applied to the acquired videos of the DUT [45]. In this regard, the main steps of the measurement procedure include: (a) loading of the video, where the frame number and frequency are evaluated; (b) identification of the instantaneous rotation center (ICR) of the comb-drive (obtained from the intersection of two lines drawn by two pairs of points selected by the operator on the first frame); (c) automatic placement of the markers within a Region of Interest (ROI) by means of the algorithms in [45]; (d) identification of the *x* and *y* coordinates of each marker over time (for each frame of the video), which made it possible to measure the angular displacement $\theta =\frac{s}{r}$ as the ratio of the arc *s* of the marker circular trajectory by its radius *r*, the latter being evaluated as the distance between the marker and the ICR. Then, velocity and acceleration have been computed as the first and second discrete derivatives of the angular displacement, respectively.

### 4.3. Uncertainty Analysis

The main sources of uncertainty have been investigated, as described in some recent investigations [32,39,40,41,42,43,44].

Type A uncertainty, $\sigma_{A}$, has been calculated directly from the standard deviation of the experimental results.

Type B uncertainty, $\sigma_{B}$, has been evaluated by considering the main sources of uncertainty in the experimental setup (Table 3):

- Power supply uncertainty on amplitude, $\delta_{V}$, and on frequency, $\delta_{F}$, reported in the datasheet of the function generator.
- Power amplifier uncertainty on amplitude, $\delta_{PA}$, reported in the datasheet of the power amplifier.
- Frame time uncertainty, $\sigma_{FT}$, evaluated assuming that the time difference between adjacent frames is not constant over the time.
- Resolution uncertainty, $\sigma_{R}$. Based on [46], it has been assumed an uncertainty on the overall resolution of about 4 μm, which, in terms of standard deviation, has been evaluated as 2.3 μm, assuming a Gaussian Probability Density Function. This term also takes into account the uncertainty of the optical system, evaluated by considering the lateral resolution that depends on diffraction and the wavelength of the incident light and assumed to be 0.4 μm [47].
- Software uncertainty, $\sigma_{S}$, also depends on the frequency and the considered quantities (displacement, velocity, and acceleration).

The implemented in-house software requires that the operator manually selects two pairs of points on the first frame of the video to obtain the ICR coordinates. Therefore, this component of uncertainty has been assessed by asking 12 different observers to perform the described above operation 30 times each, for a total of 360 pairs of *x* and *y* coordinates that have been processed by a Monte Carlo simulation (MCS) with $10^{4}$ iterations. At each iteration of the MCS, two random coordinates of the ICR are generated. Finally, Type A and Type B uncertainties have been combined [48] to estimate the total uncertainty as follows:

(1) $$\sigma_{T}=\sqrt{\sigma_{A}^{2}+\sigma_{B}^{2}}$$

### 4.4. Comparison between the Simulated and the Experimental Data

Considering the entire variety of analyzed sinusoidal output periods, the difference between the measured values $x_{a}$ and the simulation data $x_{e}$ has been evaluated in terms of percentage error (PE)

(2) $$PE_{n}={\left|\frac{x_{a}-x_{e}}{x_{a}}\right|}_{n}\times 100$$

for every video frame.

## 5. Numerical Simulation

**Figure 11:**
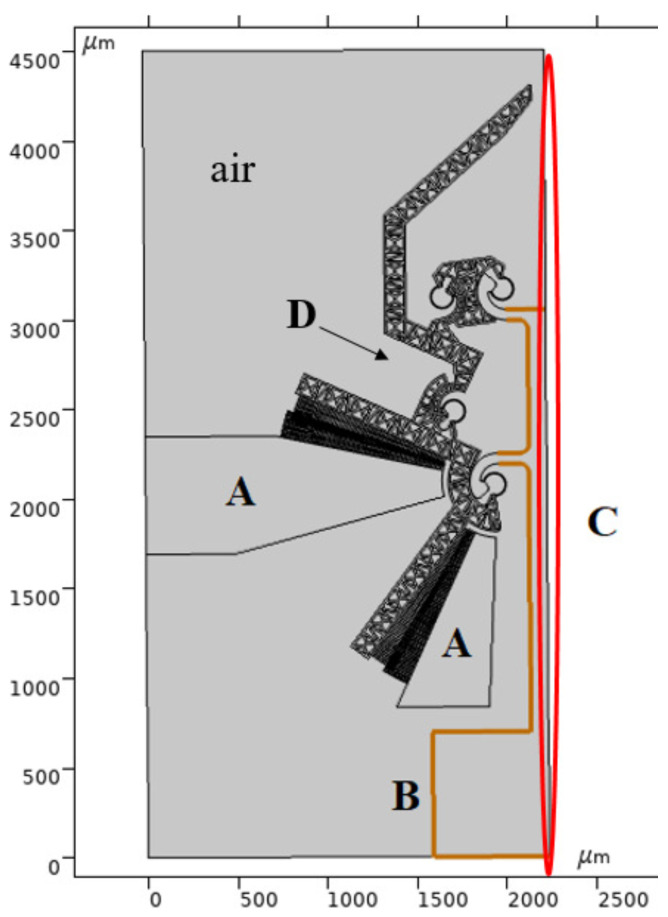
Microgripper 2D model and set-up: fixed and electrically grounded regions (A); fixed pad (B); symmetric boundary edge (C); floating parts (D).

Finite element analysis (FEA) has been adopted to numerically simulate the gripper response when an electric potential is supplied to the electrostatic comb-drives. The commercial software Comsol Multiphysics^®^ has been used to perform the numerical simulations. Symmetry boundary conditions have been conveniently exploited to reduce the computational costs, so only one-half of the microgripper has been considered (Figure 11). FEA simulations have been carried out by implementing the 2D model of the microgripper (40 μm out-of-plane thickness) to further reduce the computational efforts.

The model also considers the air, which surrounds the gripper, as a free deforming domain to properly simulate the electrostatic actuation physics. The considered mechanical and electric boundary conditions are listed below (see Figure 11):

- the non-moving fingers are anchored and electrically grounded through the ground pad (A);
- the moving fingers and the remaining gripper parts (D) can move in the plane with respect to the highlighted pad (B);
- the edge (C) represents the symmetric boundary condition.

**Figure 12:**
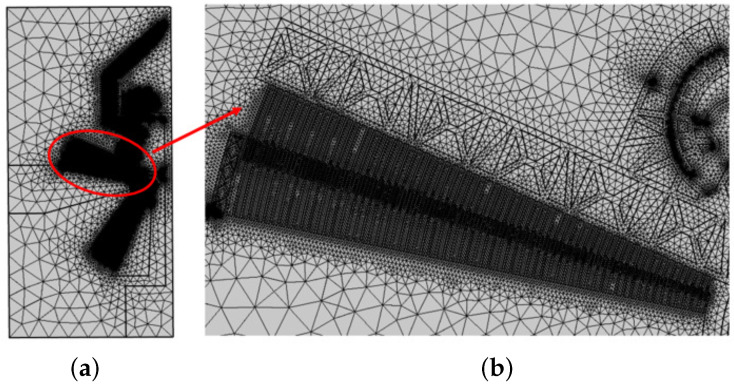
View of the overall (**a**) and local (**b**) generated mesh.

As shown in Figure 12, the overall mesh (a) has been particularly refined in correspondence to the thinnest elements and in the air domain. Figure 12b shows the details of the mesh around the region of the fingers. The total mesh size consists of 81,500 triangular elements, and the quadratic serendipity geometry shape function was selected. The microgripper is made of silicon, and the anisotropic formulation of elasticity was implemented from [49]. Furthermore, non-linearity due to large deflections has also been considered.

The key parameters in electrostatic actuation simulations are the relative permittivity of the solid and dielectric medium, as well as the elastic modulus of the moving member. In the literature, many works report different values for the Young’s modulus of silicon depending on the doping type and crystal orientation, so the range of values seems very wide (62 GPa–165 GPa, see [50]). In this work, the values of the silicon stiffness matrix were inferred from the experimental tests reported in Section 6. In particular, with respect to the values proposed by [49] for the anisotropic stiffness coefficients of Si 〈100〉, a correction factor of 0.4 was calculated. Such value is justified for several reasons:

- the actual microgripper SOI wafer stiffness matrix is unknown because it is a composite material;
- The FEA model considers constant out-of-plane thickness and ideal geometry. On the other hand, each device layer shows non-constant thickness, and geometric imperfection is unavoidable in the actual fabricated microgripper. Therefore, the flexural stiffness of the CSFHs (which mostly impacts the simulations) may randomly vary as a result of the fabrication process;
- the actual device layer presents an aluminum masking that is not considered in FEA.

**Figure 13:**
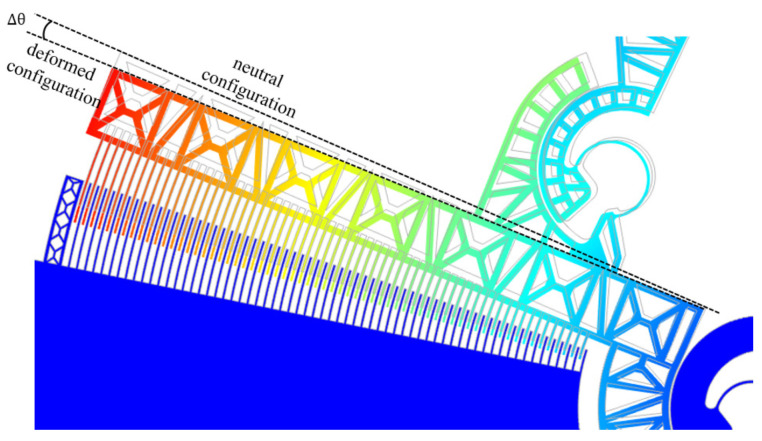
Angular displacement Δθ between the fixed and moving parts of a comb-drive.

**Figure 14:**
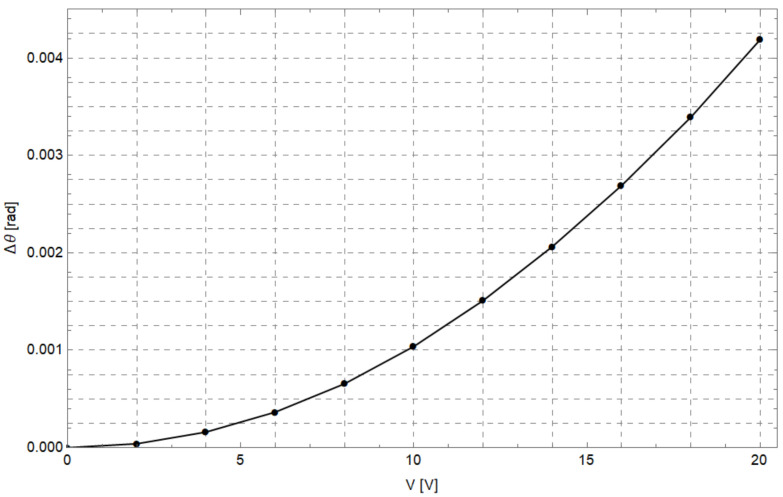
Comb-drives angular displacement Δθ vs. voltage.

**Figure 15:**
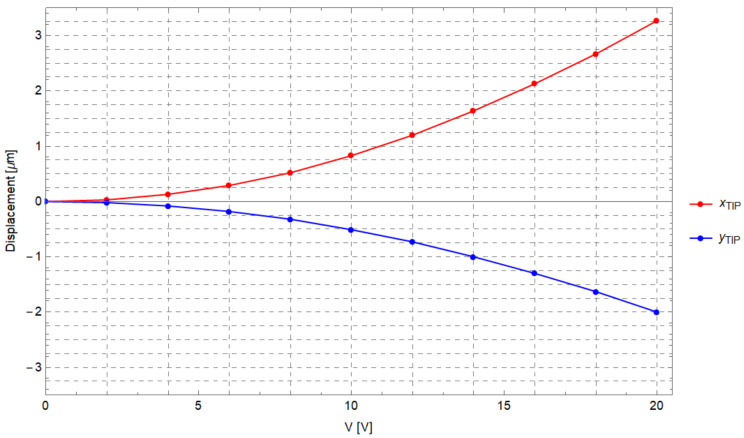
*x*- and *y*-gripper tip displacement components vs. voltage.

As a preliminary step, the angular displacements Δθ sustained by the comb-drives (see Figure 13) were simulated by varying the potential *V* applied between the fixed (A) and moving elements (D) of the comb-drives (see Figure 11). Figure 14 shows the angular displacements obtained by varying the potential between 0 V and 20 V. In addition, the *x*- and *y*-components of the gripper tip displacement have also been calculated and synthesized in the graph reported in Figure 15. The maximum *x*- and *y*-tip displacement is, respectively, 3.27 μm and −2.00 μm at V= 20 V.

**Figure 16:**
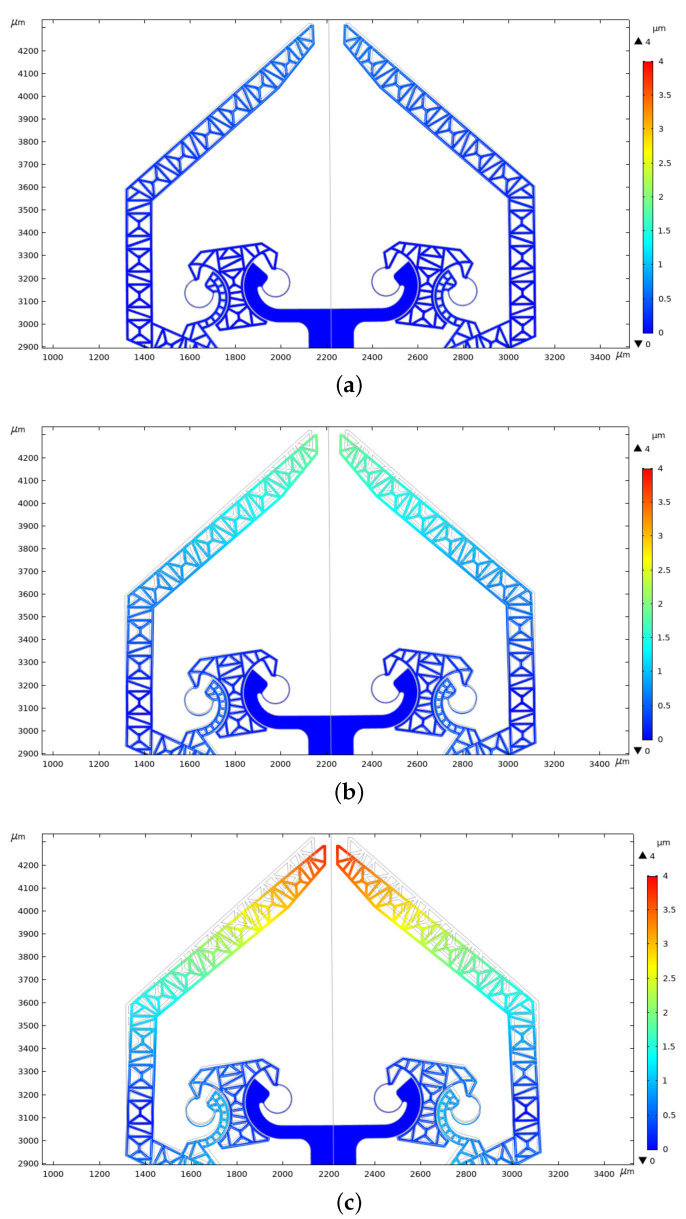
Total displacement map for different values of potential: (**a**) 8 V, (**b**) 14 V, (**c**) 20 V (as usual, the deformation map does not use 1:1 scale to magnify displacements).

The total displacements map, in the region nearby the grippers, is shown in Figure 16 where the device in the neutral configuration is depicted in gray. Significant displacements of the gripper tip appear after 10–14 V.

The dynamic response to a time-dependent potential

(3) V(t)=A+Asin(2πft)

where A=10 V and *f* is the frequency, has been numerically and experimentally investigated.

**Figure 17:**
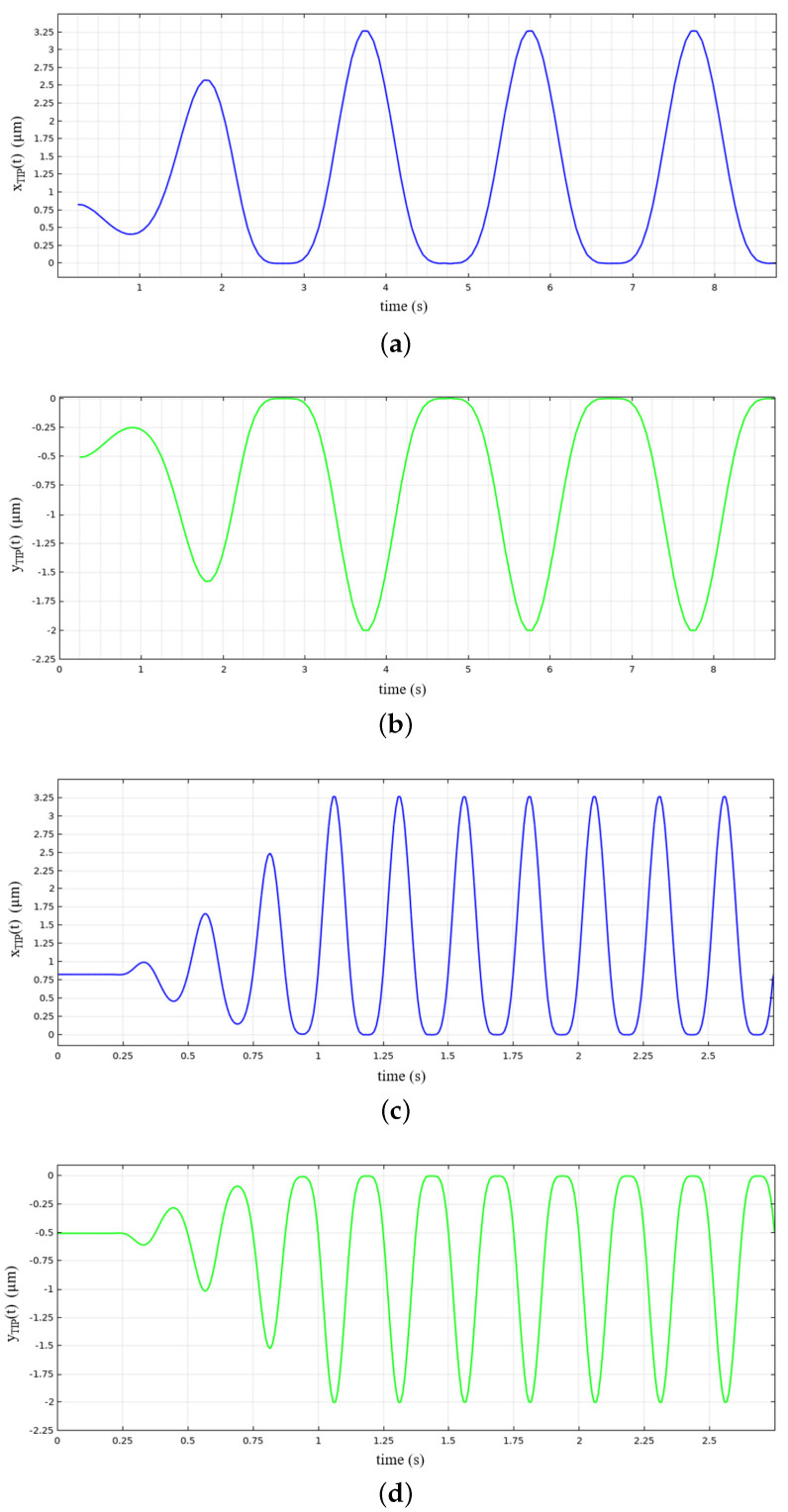
Tip displacements along *x* and *y* components vs. time: (**a**,**b**) plots have been computed for f=0.5 Hz; (**c**,**d**) plots have been computed for f=4 Hz.

Figure 17 represents the *x*- and *y*-tip displacements obtained considering 0.5 Hz and 4 Hz and shows that the tip maximum displacement, in the selected frequency range, is independent of the frequency, whereas both maxima of the *x*- and *y*-components are equal to those obtained in the case of 20 V constant potential (see Figure 15).

## 6. Results and Discussion

**Figure 18:**
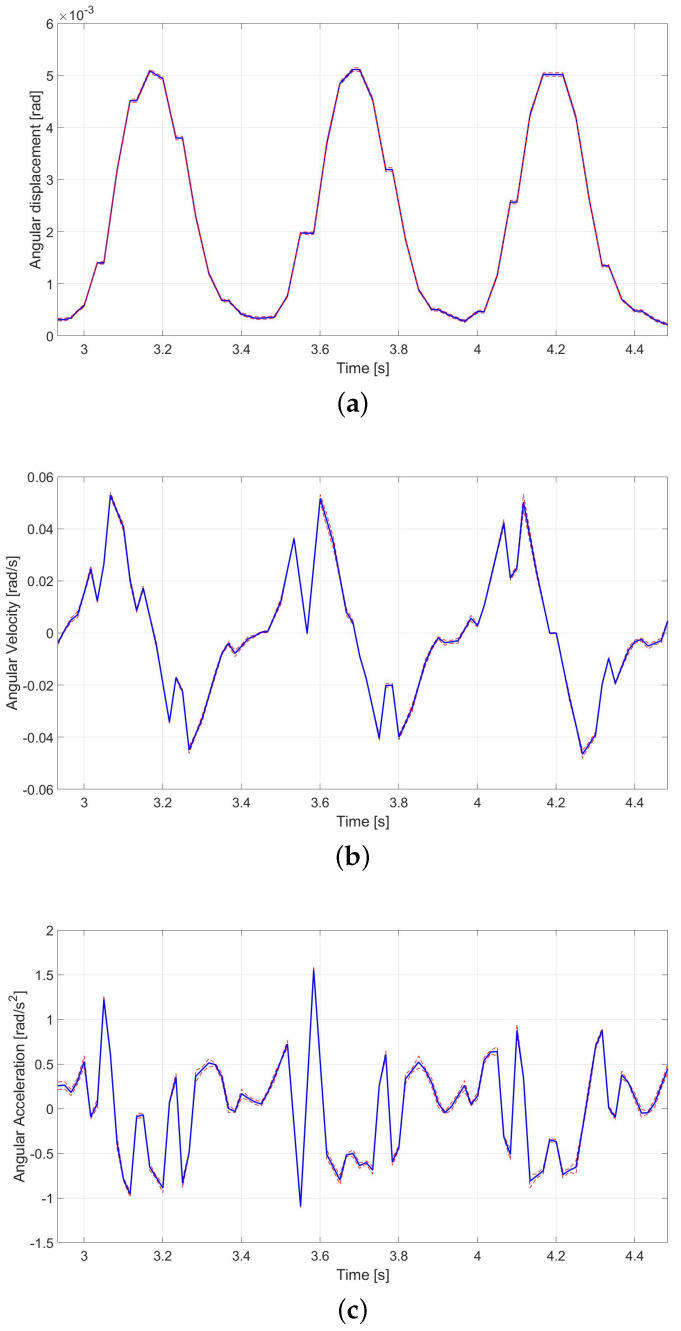
Angular displacement (**a**), angular velocity (**b**), and angular acceleration (**c**) (blue line) reported with their measurement uncertainty $\sigma_{T}$ (red dashed line) for the DUT powered at 2.0 Hz sinusoidal voltage

Figure 18 presents the comb-drive angular displacements (a), angular velocities (b), and angular accelerations (c), together with their total uncertainties that have been obtained when the DUT is powered according to Equation (3) at 2.0 Hz.

Moreover, the experimental results for all the analyzed frequencies (0.5 Hz, 1.0 Hz, 1.5 Hz, 2.0 Hz, 3.0 Hz, and 4.0 Hz) are reported in Table 4 in terms of the maximum value of the measured quantity and their estimated uncertainties (expressed as standard deviation, $\sigma_{T}$).

The values reported in Table 4 show that the total uncertainties remain below 2%, 3%, and 4% for the angular displacement (about $5\times 10^{-3}$ rad), the angular velocity (from $15\times 10^{-3}$ rad/s to $70\times 10^{-3}$ rad/s), and the angular acceleration (from $360\times 10^{-3}$ rad/s${}^{2}$ to $2230\times 10^{-3}$ rad/s${}^{2}$), respectively, for frequencies higher than 1.0 Hz.

**Figure 19:**
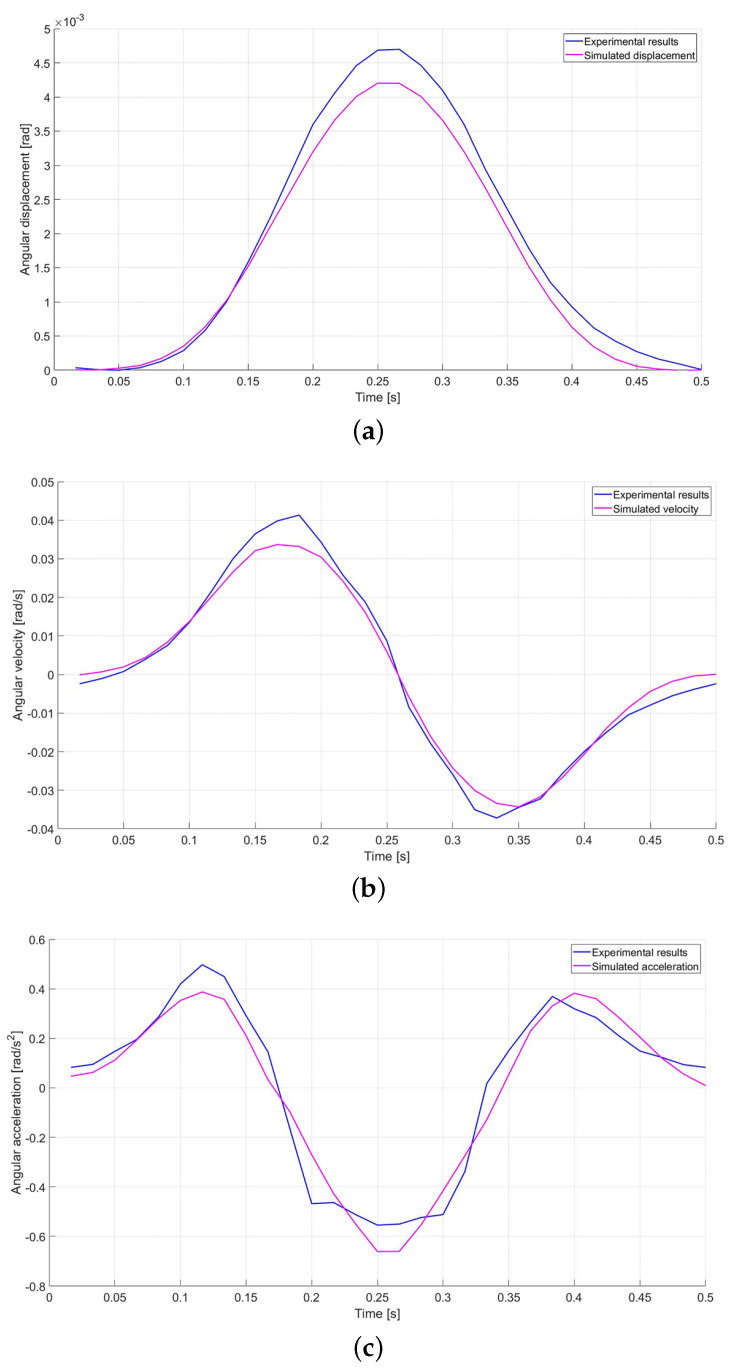
Experimental (blue) and simulated (magenta) angular displacement (**a**), velocity (**b**), and acceleration (**c**) at 2.0 Hz.

The mean over all periods of the acquired data has been calculated and compared to the value obtained by means of the numerical model. Figure 19 shows the results obtained from the comparison between the experimental data and the simulated data when DUT is powered with a sinusoidal supply frequency of 2.0 Hz.

Table 5 presents the maximum values of the percentage error PE, as expressed in Equation (2), for all the examined frequencies. This table shows that the PE values remain below 1%, 1.6%, and 3% for angular displacements, velocity, and acceleration, respectively.

**Figure 20:**
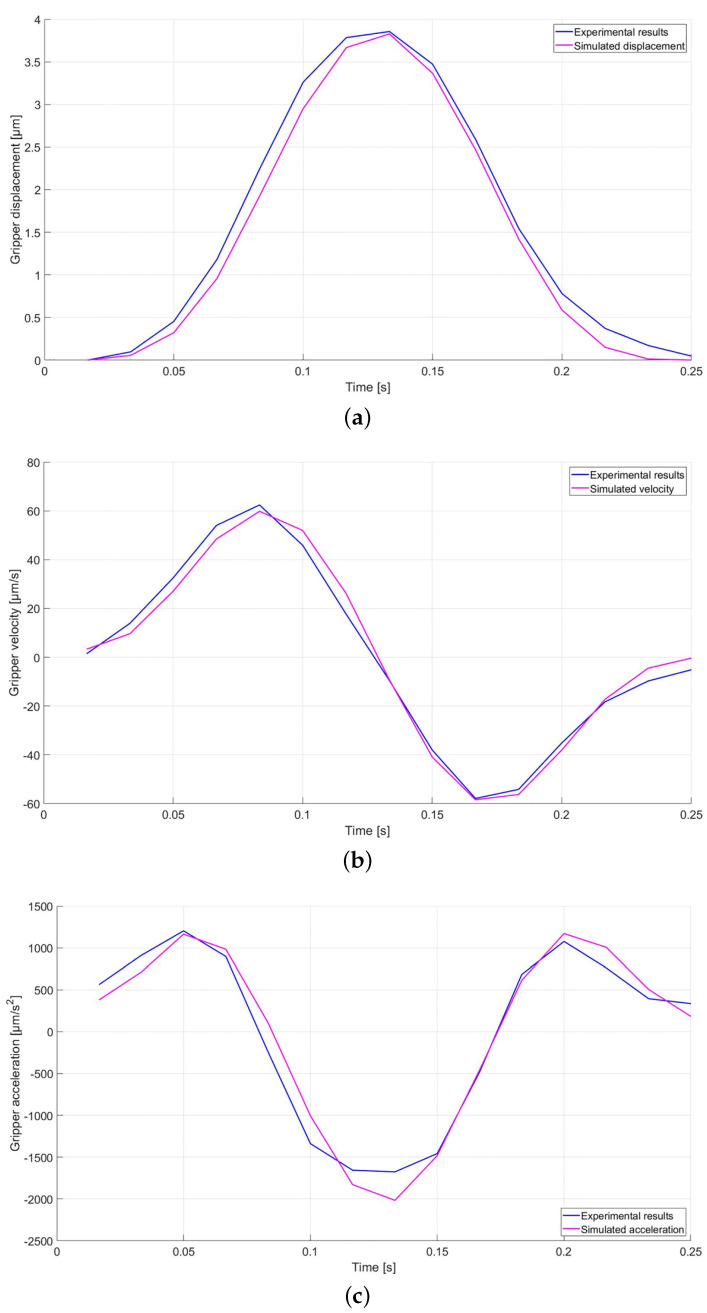
Experimental (blue) and simulated (magenta) displacement (**a**), velocity (**b**), and acceleration (**c**) at a 4.0 Hz

Furthermore, Figure 20 presents a comparison between the simulations and the experimental results for the gripper displacements, velocity, and acceleration when the DUT is powered by a sinusoidal supply signal working at a 4.0 Hz frequency, while the corresponding PE values are reported in Table 6.

The PE values presented in Table 6 have all been estimated to be less than 1% for gripper displacement, velocity, and acceleration, while other studies could be carried out to improve the accuracy of the method for very small linear displacements.

The above-presented results are quite encouraging for future applications of the proposed method, provided that they are used to calibrate the FEM model. More generally, the method can be extended to other MEMS-Technology-based devices to improve their simulation accuracy and reliability, specially in the field of biological tissues and cells dynamic manipulation, supporting designers to optimize configurations suitable for specific operational environments.

## 7. Conclusions

The idea of increasing the torque exerted by rotary-comb drives by arranging two cooperative arrays of fingers has been investigated in the present paper. The study was stimulated by the need to develop a microgripper for biological tissue and cell manipulation with a complex structure, consisting of two four-bar linkages in a mirroring configuration, with eight CSFH type flexures. This complexity has been used in the design stage to successfully assign a prescribed motion of the tips. The adopted fabrication process, based on DRIE (Deep Reactive Ion Etching) on a SOI (Silicon-on-Insulator) wafer, was able to yield some prototypes that could be tested in different dynamic experiment. A numerical simulator has been built and calibrated to serve as a tool during future experimental campaigns planned for validation and prototype demonstration in relevant environments. The proposed microgripper design turned out to be promising for low-frequency tissues and cells’ dynamic manipulation.

## Figures and Tables

**Table 1:** Comb-drive and CSFH design specifications. It is worth noting that CSFH clearance is smaller than fingers clearance to avoid finger contacts.

Component	Label	Value
Finger	Width	4 μm
	Min length	38 μm
	Max length	151 μm
	Thickness	40 μm
	Distance	10 μm
	Finger clearance	3 μm
Overlapping	Initial angle	2${}^{\circ }$
	Rotor-Stator finger distance	3 μm
SOI Wafer	Device layer thickness	40 μm
	Buried oxide layer	5 μm
	Handle layer	400 μm
CSFH	Curved beam length	252 μm
	Curved beam width	5 μm
	Curved beam thickness	40 μm
	Curvature radius	62.5 μm
	Conjugate surfaces clearance	2.5 μm

**Table 2:** Experimental setup.

Device	Characteristics
	Silicon microgripper, device thickness 40 μm,
Device Under Test (DUT)	insulated layer thickness 3 μm, handle thickness 400 μm,
	capacitive Comb-Drives.
	YOKOGAWA FG420
	Amplitude: Setting range: 0 to 10 V${}_{p-p}$,
Function Generator	Resolution: 36 mV${}_{p-p}$,
	Accuracy: ±1% of amplitude setting [V${}_{p-p}$] + 2 mV${}_{p-p}$
	KEPCO BOP 20-20D
Power Amplifier	Output: 0 to ±20 V, Accuracy: ±2 mV
	n.1 MP25L, n.1 MP25R,
Micropositioner	range X/Y/Z 10/10/10 mm
	with 5 μm resolution
Probes (supply)	PA-C-1M with tungsten needles
	NB50TS, zoom range 0.8× 5× (8×–50×),
Light Microscope	LED illumination Transmitted-Reflected,
	B2-1525 additional objective 2×
Digital Camera	MD6iS, 6MP, pixel dimension, 2.8 μm × 2.8 μm,
	maximum resolution 3264 × 1840 px
Image Processing Software	In-house software developed in MATLAB environment
	(2020b, MathWorks)
	AMD Ryzen 5 3500U
PC	with Radeon Vega Mobile Gfx 2.10 GHz,
	8.00 GB RAM

**Table 3:** Main type B uncertainty source.

Source	Value
Power supply uncertainty on amplitude $\delta_{V}$	1% of [V${}_{p-p}$] + 2 mV
Power supply uncertainty on frequency $\delta_{V}$	0.01 μHz
Power amplifier uncertainty $\delta_{PA}$	2 mV
Frame time uncertainty $\sigma_{FT}$	1 ms
Resolution uncertainty $\sigma_{R}$	2.3 μm
	it depends on frequency,
	as well as on considered quantities
Software uncertainty $\sigma_{S}$	(displacement, velocity, and acceleration)
	and on every time instant
	of the output signal.

**Table 4:** Angular displacements, velocities, and accelerations at different frequencies.

Maximum Value	Angular Displacement (rad)	Angular Velocity (rad/s)	Angular Acceleration (rad/s^2^)
0.5 Hz	$\left(5.7\pm 0.1\right)\times 10^{-3}$	$\left(1.5\pm 0.1\right)\times 10^{-2}$	0.24±0.07
1.0 Hz	$\left(5.6\pm 0.1\right)\times 10^{-3}$	$\left(2.7\pm 0.1\right)\times 10^{-2}$	0.36±0.05
1.5 Hz	$\left(5.4\pm 0.1\right)\times 10^{-3}$	$\left(3.8\pm 0.1\right)\times 10^{-2}$	0.58±0.06
2.0 Hz	$\left(5.2\pm 0.1\right)\times 10^{-3}$	$\left(4.2\pm 0.1\right)\times 10^{-2}$	0.98±0.06
3.0 Hz	$\left(4.8\pm 0.1\right)\times 10^{-3}$	$\left(5.5\pm 0.1\right)\times 10^{-2}$	1.66±0.06
4.0 Hz	$\left(4.5\pm 0.1\right)\times 10^{-3}$	$\left(6.8\pm 0.1\right)\times 10^{-2}$	2.23±0.07

**Table 5:** Percentage Error (PE) between the experimental and the simulated data.

Frequency	Angular Displacement (rad)	Angular Velocity (rad/s)	Angular Acceleration (rad/s^2^)
0.5 Hz	<1%	<1%	<3%
1.0 Hz	<1%	<1.4%	<2%
1.5 Hz	<1%	<1%	<1.5%
2.0 Hz	<1%	<1.6%	<0.8%
3.0 Hz	<1%	<0.8%	<0.5%
4.0 Hz	<1%	<0.6%	<0.5%

**Table 6:** Percentage Error (PE) between the experimental and simulated data.

Frequency	Displacement	Velocity	Acceleration
4.0 Hz	<1%	<1%	<1%
